# Simple vs. Complex Carbohydrate Dietary Patterns and the Global Overweight and Obesity Pandemic

**DOI:** 10.3390/ijerph14101174

**Published:** 2017-10-04

**Authors:** Fabrizio Ferretti, Michele Mariani

**Affiliations:** Department of Communication and Economics, School of Social Sciences, University of Modena and Reggio Emilia, Palazzo Dossetti—Viale Allegri 9, 42121 Reggio Emilia, Italy; michele.mariani@unimore.it

**Keywords:** carbohydrates, dietary patterns, human development, overweight and obesity, nutrition transition

## Abstract

Nowadays, obesity and being overweight are among the major global health concerns. Many, diet-related diseases impose high tangible and intangible costs, and threaten the sustainability of health-care systems worldwide. In this study, we model, at the macroeconomic level, the impact of energy intake from different types of carbohydrates on the population’s BMI (body mass index). We proceed in three steps. First, we develop a framework to analyse both the consumption choices between simple and complex carbohydrates and the effects of these choices on people health conditions. Second, we collect figures for 185 countries (over the period 2012–2014) regarding the shares of simple (sugar and sweetener) and complex (cereal) carbohydrates in each country’s total dietary energy supply. Third, we use regression techniques to: (1) estimate the impact of these shares on the country’s prevalence of obesity and being overweight; (2) compute for each country an indicator of dietary pattern based on the ratio between simple and complex carbohydrates, weighted by their estimated effects on the prevalence of obesity and being overweight; and (3) measure the elasticity of the prevalence of obesity and being overweight with respect to changes in both carbohydrate dietary pattern and income per capita. We find that unhealthy eating habits and the associated prevalence of excessive body fat accumulation tend to behave as a ‘normal good’ in low, medium- and high-HDI (Human Development Index) countries, but as an ‘inferior good’ in very high-HDI countries.

## 1. Introduction

There is increasing emphasis on obesity as a major global public health and economic problem [1]. According to the World Health Organization (WHO), the prevalence of obesity worldwide has more than doubled since 1980 and, today, most of the world’s populations live in countries where being overweight and obesity kill more people than being underweight [2]. It is widely acknowledged that obesity and being overweight are important risk factors for a variety of chronic non-communicable diseases (including cardiovascular diseases, diabetes, musculoskeletal disorders, and even some types of cancer) [3].

These diet-related diseases impose high tangible and intangible costs on society and threaten the sustainability of health-care systems worldwide—a problem usually exacerbated by the contemporary rising global trends in population ageing [4]. The so-called ‘obesity pandemic’ is, indeed, not restricted to advanced economies. The growth of the prevalence of obesity and being overweight is often faster in emerging economies than in the Western countries [5]. In China, for instance, the prevalence of obesity and being overweight has grown significantly during the last two decades, especially among children in urban areas, where overweight and obesity rates have increased more than ten times since 1985 [6].

Obesity and being overweight are complex health issues with a multi-factorial aetiology, involving genetic, behavioral, and environmental (i.e., cultural, economic and social) factors [7]. Despite this, comprehensive epidemiological analysis of world trends in the Body Mass Index (BMI) usually pay little attention to the economic forces that underlies the overweight and obesity pandemic [8,9]. However, research has consistently shown that the increasing prevalence of obesity and being overweight—and especially its spread in rapidly growing economies—reflects structural changes in people’s health-related habits and behaviors, which are inextricably linked with improving living standards [10,11].

The main characteristic of the conditions of obesity and being overweight is the abnormal (or excessive) accumulation of body fat resulting from an energy imbalance between two flows: the inflow of calories consumed and the outflow of calories expended [12]. In the vast majority of world cultures and geographic regions, carbohydrates usually serve as the human body’s primary source of calories [13]. However, not all carbohydrates ‘are created equal’ in nutritional sciences. Specifically, complex carbohydrates—derived from whole and unprocessed plant-based foods—are generally considered healthier than simple carbohydrates, especially those derived from high-processed and sugar-added foods and beverages—which usually provide ‘empty calories’ (i.e., calories without nutrients)—whose increasing consumption is regarded as a major driver of the worldwide spread of obesity and being overweight, notably among children and young adults [14,15,16].

In this study, we model—at the macroeconomic level—the impact of energy intake from different types of carbohydrates on the population’s BMI. To this end, we proceed in three steps. Firstly, we develop a theoretical framework to analyse both the consumption choices between simple and complex carbohydrates and the effects of these choices on consumers’ health conditions. Second, we collect figures for 185 countries (over the period 2012–2014) regarding the shares of simple (sugar and sweetener) and complex (cereal) carbohydrates in each country’s total dietary energy supply. Third, we use regression techniques to: (1) estimate the impact of these shares on the country’s prevalence of obesity and being overweight; (2) compute for each country an indicator of dietary pattern based on the ratio between simple and complex carbohydrates, weighted by their estimated effects on the prevalence of obesity and being overweight; and (3) measure the elasticity of the prevalence of obesity and being overweight with respect to changes in both carbohydrate dietary pattern and income per capita.

The remainder of the paper is structured as follows. In the next section we develop the model of consumer food choice that grounds our quantitative work. Section 3 illustrates the econometric methods and data sources. The results are presented in Section 4. Finally, Section 5 concludes with a discussion and some brief policy recommendations.

## 2. Theory

After Grossman’s seminal model of the demand for health [17], it is usual to model consumers’ behavior by combining the utility function (and the budget constraint) with a health production function that indicates the impact of the consumers’ health-related choices on their health status (measured in terms of morbidity, mortality, life expectancy, or other health-adjusted indicators of quality of life). From this approach, as shown by Wagstaff [18], a simple macroeconomic model can be derived to empirically study how changes in health-related choices (due, for instance, to changes in consumers’ preferences or income) affect individuals’ health conditions and, thus, the prevalence of health problems in a given population. In this paper, a generalization of Wagstaff’s model, recently provided by Coppola [19], is adapted to the study of the effects on BMI of different carbohydrate dietary patterns.

Let us consider a simplified economy in which there are only two types of foods: (1) low-processed foods, made with whole cereals and vegetables; and (2) high-processed foods, made with refined flours and added sugars. The former are the source of complex carbohydrates with a low glycaemic index, whereas the latter are the source of simple carbohydrates with a high glycaemic index. We assume that people consuming any positive combination of these two kinds of foods in order to meet their daily energy requirements. However, the health effects of low- and high-processed foods are different. A whole-grain- and vegetable-based diet (i.e., a diet based mainly on low-calorie and high-nutrient foods) has ‘protective’ health effects, helping people to maintain a healthy weight and decreasing their likelihood of developing diet-related chronic non-communicable diseases. Conversely, a refined-flour- and added-sugar-based diet (i.e., a diet based mainly on high-calorie and low-nutrient foods) is one of the leading causes of unhealthy weight gains and is associated with the onset of the Metabolic Syndrome, which tends to raise the risk of developing several diet-related chronic non-communicable diseases [20,21,22].

In the following, let us denote by *H* a measure of the individual’s health status. Specifically, *H* is the difference between the actual and the ideal body weight, measured according to the WHO recommended healthy BMI range [23]. Our focus is on obesity and being overweight, thus, *H* is positive or at least equal to zero (i.e., we do not deal with problems of undernourishment). As a result, the greater the value of *H* the greater is the unhealthy excess of body weight and, thus, the more severe is the individual’s overweight or obesity condition. The health effects of the individual’s eating habits are described by the following health production function:

(1) $$H=s^{\rho }\times c^{-\sigma }$$

where *s* stands for ‘sugar’ (i.e., the consumption of simple carbohydrates from high-processed foods) and *c* stands for ‘cereal’ (i.e., the consumption of complex carbohydrates from low-processed foods). Both, *s* and *c* are measured in kcal per capita per day. In Equation (1), the exponents ρ and σ measure the elasticity of *H* with respect to *s* and *c*. Since *H* measures the excess of body weight compared to set health standards, ρ and σ are, respectively, positive and negative, because the consumption of *s* worsens health by promoting obesity and being overweight, whereas the consumption of *c* improves health by protecting against the onset of obesity and being overweight. For these reasons, hereafter, we will refer to *c* and *s* simply as healthy and unhealthy food, respectively.

The dimension of ρ and σ is ultimately determined by the functioning of the metabolic processes that underlie the physiology of human nutrition. However, these coefficients may be manipulated by the agro-food industry. Such manipulations are constrained by the available technology and the country’s regulatory framework, and may have either positive or negative effects on consumers’ health. For instance, in the case of *s*, the agro-food industry can operate by adding sweeteners, such as the high-fructose corn syrup, so as to increase the food palatability, but worsening its adverse health effects (and, thus, raising the value of ρ).

In this simplified economy, people have preferences regarding food consumption and health conditions [24]. These preferences are described by the following Cobb–Douglas utility function:

(2) $$U=c^{\alpha }\times s^{\beta }\times H^{-\eta }$$

where *U* is the consumer’s utility (i.e., the total satisfaction that results from any given combination of goods and health status). In Equation (2), the parameters α, β, and η are elasticity coefficients that measure the reactivity of *U* with respect to *c*, *s*, and *H*. The value of these coefficients depends on individual preferences and, thus, it changes from one consumer to another.

Specifically, regarding food consumption, we assume that Equation (2) has the usual property of positive, but diminishing, marginal utility (i.e., *c* and *s* are ‘goods’, whose consumption increases the total utility but at a decreasing rate (0 < α < 1 and 0 < β < 1). In the case of health, we assume, instead, that η is negative, because the condition of being overweight or obese is a ‘bad’ (i.e., something that confers disutility). In other words, if η were equal to zero the consumer would not care about being overweight or obese. On the contrary, η < 0 implies that we are assuming health-conscious consumers who, at least partially, are concerned about their health conditions. This can be explained by the awareness of the negative effects of obesity and being overweight on future health status (i.e., the increasing risk of developing some overweight and obesity-related diseases) and/or by the cultural and social pressure to conform to a stereotype of an ‘ideal body shape’ (e.g., the social pressure on girls and young women to be thin).

Equations (2) and (3) define a framework in which the population’s health conditions, measured by the number of individuals that are overweight or obese, depend on people choices as consumers. The usual population’s eating habits determine a general profile of food consumption—i.e., a dietary pattern—that affects the prevalence of obesity and being overweight. This can be shown by replacing *H* in the utility function (Equation (2)) with its expression from the health production function (Equation (1)), as follows:

(3) $$U=c^{\alpha \;+\;\sigma \eta }\times s^{\beta \;-\;\rho \eta }$$

This expression shows that the individuals’ eating habits are a function of three fundamental factors: (1) the consumers’ tastes and preferences regarding the different kinds of foods available in the market (that is, the elasticity of utility with respect to *c* and *s*, measured by the parameters α and β); (2) the role that health plays in the individuals’ preferences, captured here by the importance of not being overweight or obese (thus, the elasticity of utility with respect to *H*, measured by the parameter η); and, finally, (3) the size of the (positive or negative) effects on people’s health of the consumption of the healthy and unhealthy foods (that is, the elasticity of *H* with respect to *c* and *s*, measured here by the parameters ρ and σ).

Health-related choices, besides preferences, also depend on the consumer’s purchasing power. We describe the set of consumer’s feasible consumption bundles by the following standard budget constraint: *p*_c_*c* + *p*_s_*s* = *Y*, where *p*_c_ and *p*_s_ denote the prices of the healthy and unhealthy foods respectively, and *Y* denotes the amount of money that consumer spends on foods (which we simply indicate as the consumer’s disposable income). Thus, each individual is facing a constrained optimization problem: to maximize utility from the consumption of goods *s* and *c* subject to the constraint of a given amount of income to spend (*Y*) and the food prices (*p*_c_ and *p*_s_), that is: *max_c_*_,s_ *U*(*c*, *s*) = *c*^α + σ^^η^ × *s*^β−ρ^^η^ subject to *p*_c_*c* + *p*_s_*s* = *Y*.

By optimally solving this problem, we obtain the consumer’s demand functions for the unhealthy and healthy food:

(4) $$Q_{S}=\frac{\beta -\rho \eta }{\alpha +\beta +\eta (\sigma -\rho )}\times \frac{Y}{p_{S}}$$

(5) $$Q_{C}=\frac{\alpha +\sigma \eta }{\alpha +\beta +\eta (\sigma -\rho )}\times \frac{Y}{p_{C}}$$

where the first term on the right-hand side of each equation is the share of the consumer’s income spent on *s* and *c* (i.e., *p*_c_*c*/*Y* and *p*_s_s/*Y*, respectively). Hence, by denoting these expenditure shares with *X*_S_ and *X*_C_, Equations (4) and (5) become *Q*_S_ = *X*_S_ × (*Y*/*p*_s_) and *Q*_C_ = *X*_C_ × (*Y*/*p*_c_), respectively. Finally, if we substitute these last two expressions—which indicate the composition of the optimal consumption bundle (i.e., the quantity demanded of *s* and *c*) given the consumer’s disposable income and the food market prices—in Equation (1), we obtain the level of overweight or obesity (*H*) as a function of the variables that drives the demand for the healthy and unhealthy food:

(6) $$H={\left(X_{S}\times \frac{Y}{p_{S}}\right)}^{\rho }\times {\left(X_{C}\times \frac{Y}{p_{C}}\right)}^{-\sigma }$$

which, by factoring out prices and income, we can more usefully rewrite as follows:

(7) $$H=(X_{S}^{\rho }\times X_{C}^{-\sigma })\times (\frac{p_{C}^{\sigma }}{p_{S}^{\rho }})\times Y^{\rho -\sigma }$$

Equation (7) shows that the excess of body fat accumulation depends on four fundamental factors, namely: (1) the share of disposable income that consumers spend on unhealthy and healthy foods (i.e., *X*_S_ and *X*_C_, respectively); (2) the food market prices (i.e., *p*_c_ and *p*_s_); (3) the consumers’ disposable income (*Y*); and, finally, (4) the elasticity of the body weight accumulation with respect to *s* and *c* (i.e., the parameters of the health production function, ρ and σ). It is worth noting that the exponent of the disposable income—the difference between ρ and σ (that hereafter we will denote by θ = ρ − σ)—may be either positive (ρ > σ) or negative (ρ < σ). This means that, by increasing the average income, the economic development may push people towards a healthier or unhealthier dietary pattern. As a result, in each population the prevalence of health problems due to obesity and being overweight may be mitigated or exacerbated by an increasing living standard. Testing the nature of this relationship is the main purpose of the next section.

## 3. Methods and Data

The central question in which we are interested is the relationship between unhealthy body fat accumulation, carbohydrate dietary patterns, and living standards. Moreover, given the hierarchical nature of the consumers’ nutritional needs and wants, variations in food prices may only anticipate or postpone demand-side structural changes that result from the overcoming of rising income threshold levels, beyond which consumers modify their health-related lifestyles [25,26]. We can therefore simplify Equation (7) by rewriting it as a relationship between three key variables: the condition of being overweight or obese (*H*), the consumption of healthy and unhealthy food (*X*_C_ and *X*_S_), and the disposable income (*Y*), as follows:

(8) $$H=(X_{S}^{\rho }\times X_{C}^{-\sigma })\times Y^{\rho -\sigma }$$

to emphasize the role of the standard of living—captured here by the level of disposable income—in determining the prevalence of obesity and being overweight. Finally, because in this model consumers spend all income on food, and both *s* and *c* are measured in terms of energy (i.e., in kcal/person/day), in Equation (8) the variables *X*_S_ and *X*_C_ directly measure the shares of the total consumers’ energy intake derived from simple and complex carbohydrates, respectively.

Our quantitative analysis is conducted at the macroeconomic level, by using cross-section data for 185 countries worldwide, over the period 2012–2014. Data come from three main sources: the Food and Agriculture Organization (FAO), the WHO, and the United Nations Development Report (UNDR). Specifically, we collected figures from:

- the FAO database [27], about the population mean consumption of simple and complex carbohydrates. These figures measure, in percentage, the shares of sugar and sweeteners (*X*_S_) and cereals (*X*_C_) in each country Dietary Energy Supply—(DES), the DES is an indicator of the total amount of food, usually expressed in kcal/person/day, available in a given country and year for human consumption;
- the WHO database [3], regarding the age-standardized adjusted rates of overweight and obesity prevalence, measured in each country by the percentage of adults (ages 20+) who have a BMI (kg/m^2^) greater than 25 (overweight, *H*_OW_) or greater than 30 (obese, *H*_OB_);
- and, finally, the UNDR database [28], concerning the level human development and that of average income, measured in each country respectively by the Human Development Index (HDI) and the Gross National Income per capita (Y, adjusted for purchasing power parity);

Table 1 contains a brief description of each variable, as well as basic summary statistics. The full database is available in the Supplementary material section (Tables S1–S3).

We, thus, proceed in three steps. First, by combining data on *X*_S_ and *X*_C_ with figures on *Y*, we estimate Equation (8) in order to measure the values of the parameters ρ and σ. For such a purpose, we use a double-log specification—that is, ln*H_i_* = β_0_ + β_1_ln*X*_S*i*_ + β_2_ln*X*_C*i*_ + β_3_ln*Y_i_* + ε*_i_*, where the coefficients β_1_, β_2_, and β_3_ measure the parameters ρ, σ, and θ = ρ − σ, respectively—by running two regressions, one for the prevalence of overweight (*H*_OW_) and the other for that of obesity (*H*_OB_). Second, we use the estimated coefficients ρ and σ to compute, for each country, an indicator of the population carbohydrate dietary pattern (that is, a carbohydrates dietary pattern index, *CDP*), based upon the ratio of the shares of simple to complex carbohydrates in the country’s DES, in which each share is weighted by the value of its negative or positive impact on health conditions:

(9) $$CDP_{i}=\frac{\rho X_{S_{i}}}{\sigma X_{C_{i}}}\times 100$$

where *i* stands for the *i*th country, and which we use as a basic measure of unhealthy eating habits, since its value increases the greater the share of calories from unhealthy foods (*X*_S_), and the greater the negative impact on health of the unhealthy food (ρ), and vice versa for *X*_C_ and σ. Third, and finally, we estimate an equation in which the prevalence of obesity and being overweight in each country is regressed against the population’s *CDP* index and the average income per capita, by using a double-log quadratic model—that is, ln*H_i_* = β_0_ + β_1_ln*DPI_i_* + β_2_ln*Y_i_* + β_3_ln*Y_i_*^2^+ ε*_i_*—which allows for a non-constant income elasticity of *H*_OW_ and *H*_OB_ with respect to *Y*.

## 4. Results

The regression results are collected in Table 2 and Table 3. Table 2 reveals that the shares of simple and complex carbohydrates in the total population’s *DES* have a significant impact on the country’s prevalence of obesity and being overweight. In both equations, the adjusted *R*^2^ is around 0.5, all coefficients have the expected sign and are statistically significant (*p* < 0.001). Specifically, our model predicts that a 1% increase in the share of sugar and sweeteners, on average, increases the country’s overweight rate by 0.3% and the obesity rate by 0.5%. On the contrary, a 1% increase in the share of cereal in the country’s *DES*, on average, decreases the overweight rate by 0.2% and the obesity rate by around 0.4%. It is worth noting that the theoretical model finds full empirical support in the results of the econometric analysis. As predicted by Equation (7), the coefficient of disposable income (ρ − σ = θ) is indeed almost equal to the algebraic sum of the coefficients of the shares of simple (*X*_S_) and complex (*X*_C_) carbohydrates (0.30 − 0.21 ≈ 0.12 and 0.53 − 0.38 ≈ 0.16, for *H*_OW_ and *H*_OB_, respectively).

Along with each country’s values of *X*_S_ and *X*_C_, the estimated coefficients ρ and σ were then used to compute the *CDP* index, according to Equation (9). We obtained two indicators: one, *CDP*_OW_, for overweight and the other, *CDP*_OB_, for obesity, because for a given *X*_S_/*X*_C_ ratio, the impact of dietary composition on the prevalence of overweight is slightly different from that on obesity. The results of these calculations are collected in Table 3 where, for ease of exposition, the average value of the *CDP* index in four groups of countries is shown, clustered according to their level of human development (measured by the HDI). The average value of the *DPI* index increases with the country’s human development, by varying from a minimum of about 15 in low-HDI countries to a maximum of about 60 in very high-HDI countries. It should be noted, however, that there is great variability within each HDI group (in the very high-HDI countries, for instance, the US index is around four times greater than that of Slovenia).

**Figure 1:**
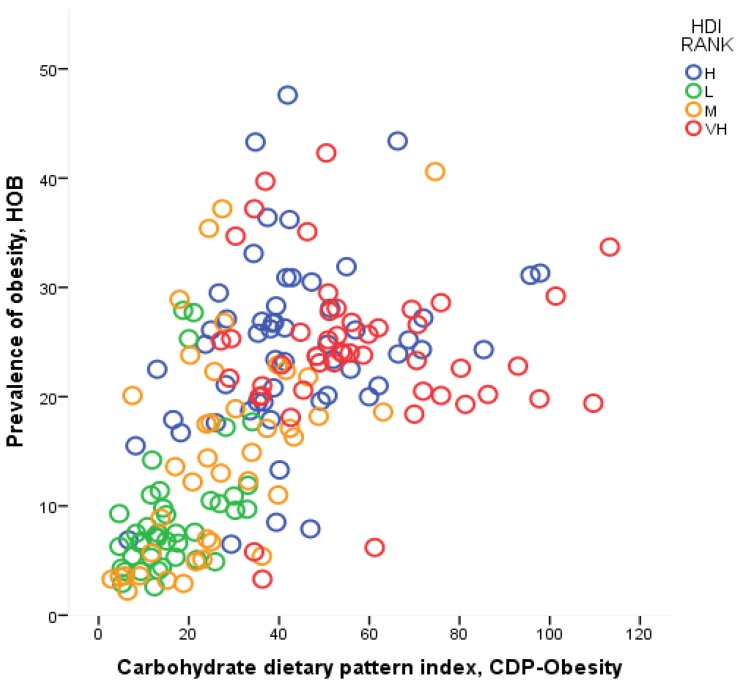
Carbohydrate dietary pattern (CDP) index and the rate of prevalence of obesity.

Table 4 contains the results of the econometric analysis in which *H*_OW_ and *H*_OB_ are regressed against *CDP* and *Y*. We can see that the model explains more than a half of the cross-country variation in the age-standardized overweight and obesity rates (here the adjusted *R*^2^ is equal to 0.58 and 0.53, respectively), and all coefficients are statistically significant (*p* < 0.001 for *DPI* and *Y*, and *p* < 0.005 for *Y*^2^). The output elasticity of both obesity and being overweight with respect to the dietary pattern (hereafter, *EHD*_OW_ and *EHD*_OB_) is positive, but less than one (the value of these coefficients is 0.26 and 0.47 for overweight and obesity, respectively). The strong relationship between the *CDP* index and the health outcome is evident from Figure 1, where *CDP* is plotted against the prevalence of obesity, *H*_OB_.

From the results collected in Table 4, we are also able to compute the income elasticity of *H*_OW_ and *H*_OB_—calculated as β_2_ + 2β_3_ln(*Y*), and hereafter denoted by *EHY*_OW_ and *EHY*_OB_, respectively. As shown in Table 5, it appears that there is a strong negative relation between these elasticities and the level of human development. Overall, the value of *EHY*_OW_ and *EHY*_OB_ is close to zero, or even negative, in very high- and high-HDI countries, and it increases with the decrease of the country’s HDI, until becoming equal, on average, at 0.25 and 0.37 in low-HDI countries for overweight and obesity, respectively.

In our simplified model, the country’s prevalence of obesity and being overweight is determined by the carbohydrate dietary pattern but, at the same time, the country’s carbohydrate dietary pattern is determined by the level of income per capita. In our notation, *H* = *f*(*CDP*) and *CDP* = *g*(*Y*). Thus, by substituting the latter equation in the former, we obtain *H* = *f*[*g*(*Y*)], and by differentiating *H* with respect to *Y*—given that (∂*H*/∂*Y*)×(*Y*/*H*) measure the elasticity of health conditions with respect to income—*EHY* can be expressed as the product of two elasticities: the elasticity of obesity and being overweight with respect to the dietary pattern (*EHD*), and the elasticity of the dietary patterns with respect to income per capita (*EDY*).

In other words, the income elasticity of the population health condition can be decomposed as follows: *EHY* = *EHD* × *EDY* [29,30]. We use this latter relationship to compute the value of *EDY*; that is, to measure for each country the reactivity of the carbohydrate dietary pattern to income per capita. Such calculations yield the results collected in the last columns of Table 5. Attention is drawn to the fact that in the low HDI countries the reactivity of the carbohydrate dietary pattern to a rising living standard is on average around 1 (0.96) and 0.8 (0.79) for overweight and obesity, respectively. On the contrary, the average value of *EDY* in middle HDI countries is always less than one (around 0.5), and it tends to decrease until becoming close to zero (or negative) in the very high-HDI countries.

## 5. Discussion

The findings this paper highlight the value of providing an economic framework to support epidemiological studies. Regarding the spread of obesity and being overweight in low- and medium-HDI countries, our econometric results support the increasing concerns about the adverse effects of economic development on the ongoing processes of nutrition transition. In these regions, a rising income per capita tends to push populations towards unhealthy Western eating habits. Specifically, a 1% increase in income leads to around a 1% increase in the *CDP* index and, on average, around a 0.2% and 0.3% increase in the overweight and obesity prevalence, respectively. On the contrary, in high HDI countries, further increases in living standards do not significantly affect the population’s dietary pattern and, thus, the spread of the obesity and being overweight problems. Even more promising results come from the very high HDI countries where, on average, the income elasticity of obesity, and that of the dietary pattern, are negative, meaning that there is a positive effect on eating habits of any further improvement in per capita income.

Furthermore, the model that underlies the econometric analysis, albeit extremely simplified, emphasizes the key variables on which public health authorities should operate to tackle the spread of obesity and being overweight. Nutrition information and education programs play a prominent role in promoting healthy eating patterns. However, our model shows that, although essential to improve consumers’ awareness about their behavior, information and education may not be enough to change consolidated unhealthy eating habits. On the one hand, there is usually a delay between unhealthy eating habits and the onset of health-related diseases. On the other hand, the consumption of unhealthy high- or ultra-processed foods usually gives the consumer a high immediate (and often addictive) satisfaction. As a result, the value of the product ρη varies with consumer impatience, and it may be very low for those who have strong preference for small immediate rewards over large delayed ones (i.e., consumers with hyperbolic discount functions) [31].

In addition to this, the agro-food industry may operate to increase the value of β, either by effective advertising and marketing strategies or by developing new and powerful additives to improve the foods ‘mouth-feel’ and ‘repeat-appeal’ (or addictiveness) [32]. Indeed, as shown in Equation (3), ceteris paribus, a greater awareness of the health effects of the different kinds of foods (in our model, a greater value of η) tends to increase utility and, thus, the consumption, of healthy food (*c*), and to decrease utility and consumption of the food that promotes overweight and obesity (*s*). However, as long as the health impact of the unhealthy food—measured by the product ρη, i.e., the negative metabolic effect (ρ) times the weight conferred by the consumer to this effect (η)—is smaller than the utility that the consumer gets by eating *s* (β), even a health-conscious consumer will continue to buy and eat positive amounts of the unhealthy food (and these amounts will be greater the more the value of β is larger than that of the product ρη). Finally, eating habits are the results of complex interactions between the cultural, economic and social environment and the consumer’s preferences [10]—at both microeconomic (i.e., family and local community) and macroeconomic (i.e., country) levels. Thus, even educated and informed consumers may hardly change their eating habits if they live in an ‘obesogenic environment’ [33,34].

The findings of our work are, however, subject to at least two main limitations. First, we use a dietary pattern indicator based only on the ratio of simple to complex carbohydrates in the countries’ DES. This may lead us to underestimate the real value of *EHD*, which is the negative impact of unhealthy diets on the prevalence of obesity and being overweight. Thus, as highlighted by a recent systematic review on the relationship between food energy density and adult body weight changes [15], further research is needed to develop an indicator able to better capture the complex health effects of high- and ultra-processed foods, which usually combine added sugars with harmful saturated fats and other unhealthy nutritional features (e.g., low fiber, high sodium, and artificial preservatives) in high calorie-dense products.

Second, in our quantitative analysis there is a lack of data on income distribution and all other cultural, economic, and social factors that affect people’s eating habits. Poorer and less educated individuals in high HDI countries, for instance, may behave more as the typical consumer of a medium-HDI country, rather than as their wealthier and more educated fellow citizens; or to take another example, gender discrimination due to religious beliefs may distinguish between eating behaviors of populations with otherwise similar levels of economic development. More comprehensive research should, thus, include new variables in both the health production function and the food demand function (including variables on each country’s food regulatory framework and food and nutrition public policy and advocacy).

## 6. Conclusions

In summary, the impact of the global nutrition transition on the overweight and obesity pandemic is a well-established research area that has allowed epidemiologists to link populations’ changing eating habits and health outcomes [35]. Although a number of different factors have been identified as key drivers of the more recent transition from healthy to unhealthy dietary patterns, the crucial role of economic development is relatively understood, especially in its quantitative dimension. In this paper we presented an economic framework in which we examine consumer behavior towards healthy and unhealthy foods in the context of increasing disposable income. Our findings corroborate previous research about the causal relationship between the ongoing processes of nutrition transition in the worldwide spread of diet-related diseases, and may help public health scholars in improving effective programs to tackle obesity and being overweight, especially in low and medium HDI countries.

## Figures and Tables

**Table 1:** Summary of statistics.

Variable	Description	Mean	Median	Max	Min	Std. Dev.	***n***
**HDI**	Human Development Index	0.697	0.727	0.949	0.352	0.155	185
**Y**	Gross National Income (GNI) per capita	16,867.1	10,382.7	129,915.6	587.5	18,521.1	185
	2011, PPP $						
**H_OW_**	Prevalence of overweight (population aged 18+)	47.7	54.8	79.3	14.5	17.0	185
	Age-standardized rate, both sex						
**H_OB_**	Prevalence of obesity (population aged 18+)	19.0	20.1	47.6	2.2	10.2	185
	Age-standardized rate, both sex						
**X_C_**	Cereals	41.8	40.6	98.1	16.6	13.6	185
	Share of dietary energy supply (% DES)						
**X_S_**	Sugar and sweeteners	9.7	10.0	21.8	1.1	4.3	185
	Share of dietary energy supply (% DES)						

**Table 2:** Regression results: H_OW_ and H_OB_ regressed on X_S_, X_C_ and Y (double-log model).

Dependent Variable	Constant	Share Sugar	Share Cereal	Income	Adj. *R*^2^	*n*
		X_S_	X_C_	Y		
Prevalence of Overweight (H_OW_)	2.72	0.30 *	–0.21 *	0.12 *	0.57	185
		0.05	0.07	0.02		
		(6.52)	(−2.83)	(5.45)		
Prevalence of Obesity (H_OB_)	1.48	0.53 *	−0.38 *	0.16 *	0.50	185
		0.08	0.13	0.04		
		(6.51)	(−2.94)	(3.96)		

Note: t-statistics in brackets, * denote statistical significance at the 1% level (*p* < 0.001).

**Table 3:** Carbohydrate dietary pattern (CDP) index.

Countries by HDI Group		CDP_OW_	CDP_OB_	
Very High	Mean	59.8	57.6	
	Std. Dev.	22.3	21.5	
	Max	117.7	113.3	USA
	Min	28.2	27.1	Slovenia
High	Mean	45.3	43.6	
	Std. Dev.	19.9	19.1	
	Max	101.7	97.9	Barbados
	Min	6.9	6.6	China
Medium	Mean	28.1	27.0	
	Std. Dev.	16.1	15.5	
	Max	77.5	74.6	Kiribati
	Min	3.0	2.9	Nepal
Low	Mean	17.0	16.4	
	Std. Dev.	8.8	8.5	
	Max	35.4	34.1	Swaziland
	Min	4.8	4.6	Benin

**Table 4:** Regression results: H_OW_ and H_OB_ regressed on CDP index and Y (double-log quadratic model).

	Constant	Carbohydrate Dietary Pattern CDP Index	Income	Income^2^	Adj. *R*^2^	*n*
**Dependent Variable**			**Y**	**Y^2^**		
Prevalence of Overweight (H_OW_)	−1.65	0.26 *	0.88 *	−0.04 **	0.58	185
		0.04	0.24	0.01		
		(7.09)	(3.67)	(−3.15)		
Prevalence of Obesity (H_OB_)	−6.00	0.47 *	1.43 *	−0.07 **	0.53	185
		0.06	0.42	0.02		
		(7.14)	(3.38)	(−3.01)		

Note: t-statistics in brackets, * and ** denote statistical significance at the 1% and 5% levels (*p* < 0.001 and *p* < 0.005).

**Table 5:** Output and Income Elasticities.

Countries by HDI Group		Overweight			Obesity			
		EHY	EHD	EDY	EHY	EHD	EDY	
**Very High**	Mean	0.003	0.266	0.010	−0.048	0.471	−0.102	
	Std. Dev.	0.036		0.135	0.061		0.128	
	Max	0.076		0.285	0.075		0.159	Montenegro
	Min	−0.103		−0.389	−0.226		−0.480	Qatar
**High**	Mean	0.091	0.266	0.341	0.100	0.471	0.212	
	Std. Dev.	0.034		0.129	0.057		0.122	
	Max	0.166		0.623	0.226		0.480	Tonga
	Min	0.008		0.031	−0.038		−0.081	Oman
**Medium**	Mean	0.158	0.266	0.594	0.213	0.471	0.452	
	Std. Dev.	0.048		0.181	0.081		0.172	
	Max	0.234		0.881	0.342		0.725	Nepal
	Min	0.048		0.179	0.028		0.059	Eq. Guinea
**Low**	Mean	0.255	0.266	0.960	0.377	0.471	0.799	
	Std. Dev.	0.049		0.184	0.082		0.175	
	Max	0.350		1.318	0.537		1.139	C. African Rep.
	Min	0.136		0.512	0.176		0.374	Swaziland

Note: HDI (Human Development Index) rank denotes the level (low, medium, high, and very high) of human development.

## Supplementary Materials

The following are available online at www.mdpi.com/1660-4601/14/10/1174/s1, Table S1: Source and short description of each variables, Table S2: Summary statistics, Table S3: Database.
